# A Case Study of Furunculosis following an Ayurvedic Oil Massage, Sudation Therapy, and Lessons to Learn

**DOI:** 10.1155/2024/3660064

**Published:** 2024-02-08

**Authors:** Satyajit Pandurang Kulkarni, Pallavi Satyajit Kulkarni, S. Kumar

**Affiliations:** ^1^Panchakarma Department, Manjushree Research Institute of Ayurvedic Science, Pethapur–Mahudi Road, Piplaj 382610, Gandhinagar, Gujarat, India; ^2^Affiliated to Gujarat Ayurveda University, Jamnagar, India; ^3^Agadtantra Avum Vidhivaidyak, Manjushree Research Institute of Ayurvedic Science, Pethapur–Mahudi Road, Piplaj 382610, Gandhinagar, Gujarat, India; ^4^Department of Disaster Management, Alagappa University, Karaikudi, Tamil Nadu, India

## Abstract

India has a long history of using sudation therapy and oil massage as Ayurvedic treatments. However, nothing is known about its side effects, and just two studies have identified side effects as cutaneous adverse drug reactions brought on by Ayurvedic oil massage. We are presenting the example of a 72-year-old adult man who visited our hospital and had his right knee massaged with Nirgudi oil followed by sudation therapy. Erythema, papules, itchiness, and scorching pain were some of his symptoms. However, these sensations only partially abated once we quit sudation therapy. Our investigation demonstrates that a Nirgudi oil massage or sudation causes the skin reaction associated with furunculosis. This case report illustrates the necessity of being aware of Panchakarma-related consequences and suggests that medical practitioners, patients, and product makers take into account the likelihood of such a reaction following Nirgudi oil massage and sudation therapy as a precaution.

## 1. Introduction

Massage and sudation therapy are popular treatments in Ayurveda and other traditional therapies for various disorders like arthritis, muscle pain, body aches, skin diseases, and even for health [1]. Ayurveda recommends massage with specific Ayurvedic medicated oils prepared from various herbs. The Ayurvedic physician chooses the medicated oil as per the disease conditions. The Ayurvedic oil massage is followed mainly by sudation therapy. The method and the drugs for the sudation therapy are also disease specific.

Nirgudi oil is an Ayurvedic medicated oil used frequently for massage for joint disorders. It is prepared from the herb Nirgudi (*Vitex negundo*). It is a herb found almost all over India and is efficacious in arthritis, gout, and other joint pains [2]. Nirgudi oil massage and sudation therapy are believed to be safe practices.

Furunculosis is a common skin and soft tissue infection that can lead to local or systemic complications rarely. Their early recognition and management are very important to avoid further complications. It is a primary infection of hair follicles leading to abscess [3]. It is an erythematous, tender, and firm eruption like a carbuncle. The purulent material generally comes out of a furuncle. It can occur anywhere on the body but tends to appear on the body areas exposed to friction. Mostly *S. aureus* is the pathogen causing furunculosis. Eventually, a furuncle will open through the skin spontaneously or after incision. It appears over the hair-bearing skin and is unrelated to trauma or foreign body [3]. Poor personal hygiene is a contributing factor to the development of furunculosis [4].

Previously, only two cases of Panchakarma complications have been reported as Cutaneous Adverse Drug Reactions (CADR) due to Ayurvedic oil massage [5, 6].

Wertman et al. reported furunculosis due to a pedicure footbath at a North Carolina nail salon [7].

Therefore, we report furunculosis, possibly due to the massage with an Ayurvedic medicated oil, “NirgudiTaila,” and sudation therapy. In this case study, a 72-year-old male got furunculosis over his right knee joint following a local massage and sudation therapy at our hospital. The takeaway message is to improve hygienic conditions in the Panchakarma theatres and ensure a proper history of allergy before performing Panchakarma procedures.

## 2. Case Presentation

### 2.1. Symptoms and Diagnosis

A 72-year-old man presented on 13^th^ April 2022 for low backache, right knee joint pain, and occasional body ache for two years. He was a farmer from a nearby village. The low back pain was moderate after overactivity and strain over the low back region. In addition, he experienced mild body aches in the whole body and mild to moderate pain in the right knee joint. A local physician examined and treated him with modern medicines; however, he wanted Ayurvedic treatment. So, he visited our hospital.

There was no history of injury or medication except the medication for these complaints. The patient was nondiabetic and nonhypertensive. There was no history of psychiatric, cardiac, nervous, or urinary disorders. The individual has no known history of allergies, including allergies to medications, food, or pollen (Table 1).

The examination of knee joints revealed the presence of crepitus and no edema over any of the knee joints. Any scar was absent over the knee joints. The knee joint pain was seen while sitting and lifting from the chair and was absent during walking. The general examination revealed no specific findings of the disease.

As per Ayurveda, this condition was diagnosed as “Janusandhigata Vata with Katigraha.” The following Samprapti Ghataka (Pathogenic factors) were seen.

  Dosha—Vata dominant
  Dhatu, Asthi—Majja, Mamsa
  Marga—Madhyama
  Avastha—Nirama

### 2.2. Management

We recommended local oil massage with Nirgudi oil over the right knee joint and plain sesame oil over the lower back, followed by sudation and oral Ayurvedic medicines (Table 2). The patient's written consent was obtained for an oil massage and sudation.

### 2.3. An Incidence of a Cutaneous Adverse Drug Reaction (ADR)

**Figure 1:**
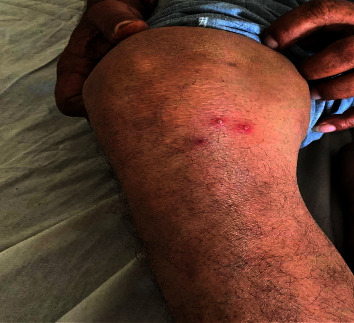
Eruptions after ayurvedic oil massage.

**Figure 2:**
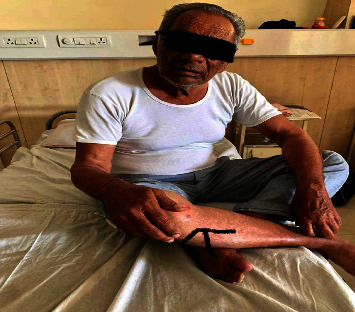
Patients showing the site of pruritus.

After massaging with Nirgudi oil over the right knee joint and sudation therapy, the red papules appeared approximately within an hour, with mild itching and redness over the knee joint. The symptoms gradually increased, with an increase in redness and burning pain (Figures 1 and 2), and after three days of consecutive massage and sudation, the burning pain and itching were unbearable; so, suspecting an allergy, we stopped the oil massage and sudation. We added an Ayurvedic medicine for allergies.

The patient was discharged and given the same medications at home. He was advised to visit after seven days but avoided it. At first, we suspected that it was an allergic reaction, but as it began to exhibit suppuration, it became clear that it was a case of furunculosis.

### 2.4. Nirgudi Oil

The text Sharangdhar Samhita mentioned this oil. The constituents of the Nirgudi oil are *Vitex negundo* and sesame oil (*Sesamum indicum*).

#### 2.4.1. Preparation of the Medicated Oil

Our staff collected the raw material needed to prepare the medicated oil in the pharmacy attached to our hospital. It is a usual practice in our hospital that we prepare the required Ayurvedic medicated oils in the pharmacy attached to our hospital.

#### 2.4.2. Sudation Therapy

We advised Swedana (sudation therapy) to the whole body for multiple joint pain and restricted movements. Immediately after the massage, the patient was asked to sit inside the sudation box. The sudation box is a specially made wooden box attached to a pressure cooker inside which Ayurvedic herbal decoction is boiled. The endpoint of this procedure was perspiration all over the body. It took less than 10 minutes for this patient.

#### 2.4.3. Assessment Parameters

The patient's main concerns were pain in their right knee joint and lower back, which were treated with Ayurvedic oil massage and sudation. However, after three consecutive days of massage, the patient experienced burning pain, eruption, and severe itching over the right knee joint. As a result, massage and sudation were stopped, and Ayurvedic medicines were prescribed to address a possible allergic reaction. The patient requested to be discharged.

We used a self-reported visual analogue scale (VAS) for their right knee joint and lower back pain to assess the patient's pain levels. We compared the VAS score from the first day to the sixth day and measured the severity of itching using the VAS scale from the fourth day to the sixth day. The VAS scale ranged from 0 to 10, with 10 indicating severe pain or itching and 0 indicating no pain or itching.

## 3. Observations

Results showed that on the first day, the patient's right knee joint pain was at a level of 6, which increased to 7 on the sixth day. In contrast, the patient's lower back pain decreased from 5 on the first day to 4 on the sixth day. The VAS scale also showed a decrease in itching severity, dropping from 7 on the fourth day to 4 on the last day.

## 4. Discussion

Panchakarma therapies in Ayurveda are in more demand as Ayurveda gains popularity in India and worldwide. Panchakarma treatments can be used to treat illnesses and promote good health. Even though the term “Panchakarma” only refers to five treatments, many allied procedures (about 25–30) have been carried out in the Panchakarma theatres. Among these, Panchakarma preoperatives include massage and sudation therapy. As a result, practically every patient receiving Panchakarma performs them. There is a lot of discussion about Panchakarma's beneficial effects. Panchakarma problems have not been documented, though.

Three cases of furunculosis caused by *M. bollettii* or *M. masiliense* due to pedicure footbaths were reported by W. Rebecca and M. Melissa. All three patients had recently undergone pedicure footbaths at a nail parlor in North Carolina. Footbaths taken before pedicures were determined to be caused symptom of infection by the authors. In our scenario, the Panchakarma theatre can be a reservoir for an infection that causes furunculosis [7].

In a community in Alaska, L.G. Michael, M.J. Barbara, A.D. Elvin, and others reported a boil outbreak. An environmental culture evaluation and case-control research were conducted in this cohort study of a village. One family was interviewed from each of the 92 homes or 77 of them. Residents who did not have boils served as the control group.

The study concluded that steam baths were linked to boil breakouts and recommended wearing a towel in a steam bath. A steam bath is performed during sudation therapy in the Panchakarma theatre. The creation of steam using Ayurvedic concoctions is the only distinction [8].

Ayurvedic oil adverse medication effects on the skin have been documented [7] and verified using patch tests. As a result, one of the difficulties of Panchakarma that must be addressed is allergies.

Patients, therapists, and consultants who practice Ayurveda should be aware of any possible hazards associated with Panchakarma therapy. Several lessons came from this situation. First, we need to keep the Panchakarma theatre clean and hygienic. Before the operation, we must obtain a thorough history of any allergies to Ayurvedic oil. Third, we must provide patients with information about the dangers and advantages of Panchakarma therapies and only administer them with their permission. Fourth, before beginning Panchakarma treatments, the written consent form needs to be modified.

## 5. Conclusion

This case study highlights the significance of Panchakarma-related consequences, which are uncommon and typically mild but can result in new diseases. To reduce the possibility of infection spreading through Panchakarma theatres, further cleanliness and hygiene should be kept. To preserve the doctor-patient relationship, it may be beneficial to adopt a positive mindset when dealing with potential complications of Panchakarma, such as furunculosis or an allergic reaction to Ayurvedic oils. In addition, it is important to educate the patient about these risks.

## Figures and Tables

**Table 1:** Timeline.

Year	Complaints
2020	The patient experienced low back and right knee joint pain and occasional body ache. He approached a local physician and was diagnosed with pain due to overactivity. Nonsignificant findings in the investigations.
2022	Due to relapse, he approached our hospital to seek Ayurvedic medicines. But he got an adverse drug reaction to Nirgudi oil massage during a six-day hospital stay.

**Table 2:** Treatment.

Procedure/drug name	Contents	Dose and duration
Nirgudi oil massage and sudation—right knee joint low back	Nirgudi, sesame oil Plain sesame oil	80 ml, 36 minutes
Dashmool Kwatha	*Aegle marmelos*, *Clerodendrum phlomidis*, *Desmodium Gmelina arborea*, *Oroxylum indicum*, *Solanum indicum*, *Solanum xanthocarpum*, *Stereospermm suaveolens*, *Tribulus terrestris*, and *Uraria picta*	10 ml orally two times after food
Sinhanad Guggul	*Commiphora mukul*, sulfur, *Terminalia chebula*, *Terminalia bellirica*, *Emblica officinalis*, and oil of *Ricinus communis*	250 mg orally two times after the food
Ashwagandha powder	*Withania somnifera*	5 gm with cow's milk two times after the food
Haridra powder	*Curcuma longa*	2 gm twice a day, two times after food

## Data Availability

No data were used in this study.

## Additional Points

*Key Messages*. Nirgudi oil massage and sudation can cause furunculosis. The administrators of the Panchakarma unit must maintain cleanliness and hygiene. The physician should consider this possibility and mention it in the written consent form (WCF) from the patient obtained before performing the procedure.
